# Novel Software for Pain Drawing Analysis

**DOI:** 10.7759/cureus.20422

**Published:** 2021-12-14

**Authors:** Asimakis K Kanellopoulos, Emmanouil K Kanellopoulos, Zacharias Dimitriadis, Nikolaos S Strimpakos, Andriana Koufogianni, Anthi A Kellari, Ioannis A Poulis

**Affiliations:** 1 Department of Physiotherapy, University of Thessaly, Lamia, GRC; 2 Biomedical Engineering, Santesoft LTD, Nicosia, CYP

**Keywords:** pain software, central sensitization, pain distribution, pain drawing, pain assessment

## Abstract

Introduction

Pain drawings (PDs) are an important component of the assessment of a patient with pain. Although analog pain drawings (APDs), such as pen-on-paper drawings, have been extensively used in clinical assessment and clinical research, there is a lack of digital pain drawing (DPD) software that would be able to quantify and analyze the digital pain distribution obtained by the patients. The aim of this work is to describe a method that can quantify the extent and location of pain through novel custom-built software able to analyze data from the digital pain drawings obtained from the patients.

Methods

The application analysis and software specifications were based on the information gathered from the literature, and the programmers created the custom-built software according to the published needs of the pain scientific community.

Results

We developed a custom-built software named “Pain Distribution,” which, among others, automatically calculates the number of the pixels the patient has chosen and therefore quantifies the pain extent, provides the frequency distribution from a group of images, and has the option to select the threshold over which the patient is considered with central sensitization (CS). Additionally, it delivers results and statistics for both every image and the frequency distribution, providing mean values, standard deviations, and CS indicators, as well as the ability to export them in *.txt file format for further analysis.

Conclusion

A novel Pain Distribution application was developed, freely available for use in any setting, clinical, research, or academic.

## Introduction

Several methods and instruments for recording the location of pain and classifying the pattern of pain have been described in the literature [1]. One method that has extensive use is pain drawings (PDs) on a body map (BM). For any pain map, the most essential property is to display topography, answering the question, “Where is your pain?” [2]. Patients draw where they feel the pain to obtain a graphic representation of pain location [3] and distribution [4].

According to the literature, PDs are used in clinical practice for various purposes, such as differential diagnosis in many pain conditions [5,6], specification of the etiology of the pain [7], or evaluation of changes in pain [8]. Additionally, PDs have been used in experimental pain studies to illustrate the location of pain induced [9-11] and predict treatment outcomes [12,13], although not always with success [14]. Several studies have been conducted to test the usability, reliability, and repeatability of PDs in chronic pain conditions [15] and demonstrated high intra-evaluator repeatability when scoring maps after one- and two-hour intervals [8].

Different instruments have been used to obtain PDs, starting from pen-on-paper drawings (analog pain drawings (APDs)) and recently developing toward digital PDs (DPDs) collected on tablet PCs [16], which offer essential advantages over the analog ones. Most European hospitals have developed digital forms to communicate and record patient data [17]. Therefore, the use of paper as a pain communication platform does not serve the aforementioned directions such as the rapid transfer and processing of data between clinicians and researchers [18].

Additionally, APDs lack an easy quantification of the results. Quantification of the pain is usually performed by circling body regions where the pain is apparent independently of the magnitude of distribution in these body areas [19]. However, this method provides a depiction of pain distribution that does not accurately reflect the pain distribution expressed as the ratio of the amount of painful area divided by the whole-body area. Although manual completion of body maps seems to be difficult to provide this type of quantification, this problem can be overcome with appropriately designed software.

Digitizing and quantifying the area of pain drawn by the patient would therefore be of high importance, especially in pain conditions such as central sensitization (CS) [20], where the topography plays a crucial part in the diagnosis. CS is defined as “an amplification of neural signaling within the central nervous system that elicits pain hypersensitivity” [21]. The diffusion of pain distribution (i.e., large pain areas with a neuroanatomically illogical distribution) is one of the basic criteria for the classification of CS pain [22]. As the expanded distribution of pain, presented as a percentage of the whole-body area, is a well-recognized sign of CS [23,24] (in consideration of the condition of the patient, its severity, and its chronicity), digital pain drawings (DPDs) would be useful in easily identifying the extended areas of pain distribution in people with conditions similar to the above.

To our knowledge, the different software used in the past to quantify PDs are either built many years ago with old software engineering technology or are not pain-oriented [25-28]. Thus, we designed and developed a freely available software to fulfill this well-established need in the literature in the pain scientific field. The aim of the present work is to describe the functions of this software and present the procedure of obtaining and analyzing data from the digital pain drawings.

## Materials and methods

The application was developed in four stages. Stages 1 and 2 were conducted by all the researchers, while stages 3 and 4 by Kanellopoulos AK and Kanellopoulos EK.

Stage 1

The literature was reviewed for the current state of DPD analysis methods and software available. The application analysis conducted was based on the information gathered from the literature, and software specifications were defined.

Stage 2

A professional illustrator created a new high-definition (HD) digital BM image (reference image (RI)). A topographical ΒΜ provides a record that is easy to read [1] and is inexpensive and can be done as often as needed, allowing for changes to be documented and the patient evaluated. However, to our knowledge, there is no single body map that is used by clinicians and researchers. Examples of different body maps are numerous, so many that it is hard even to cite them, making comparisons between patients and between conditions difficult, if not impossible. Τhe preliminary RI layouts were assessed by the whole team, and the final RI was developed.

Stage 3

The programmers created the preliminary screen layouts, which were assessed by the whole team according to the needs defined in stage 1. Based on the results, the final layout of the screens and procedures were developed. The programmers finalized the custom-built software, developed in VC++ and designed to use exclusively our RI in order to automate its calculations.

Stage 4

The application was tested thoroughly for validity and reliability errors (bugs) until its performance became bug-free.

## Results

Novel digital BM image and analysis software were created from scratch in an attempt of fulfilling the needs of both clinicians and researchers in the field of pain, with the following technical characteristics.

**Figure 1:**
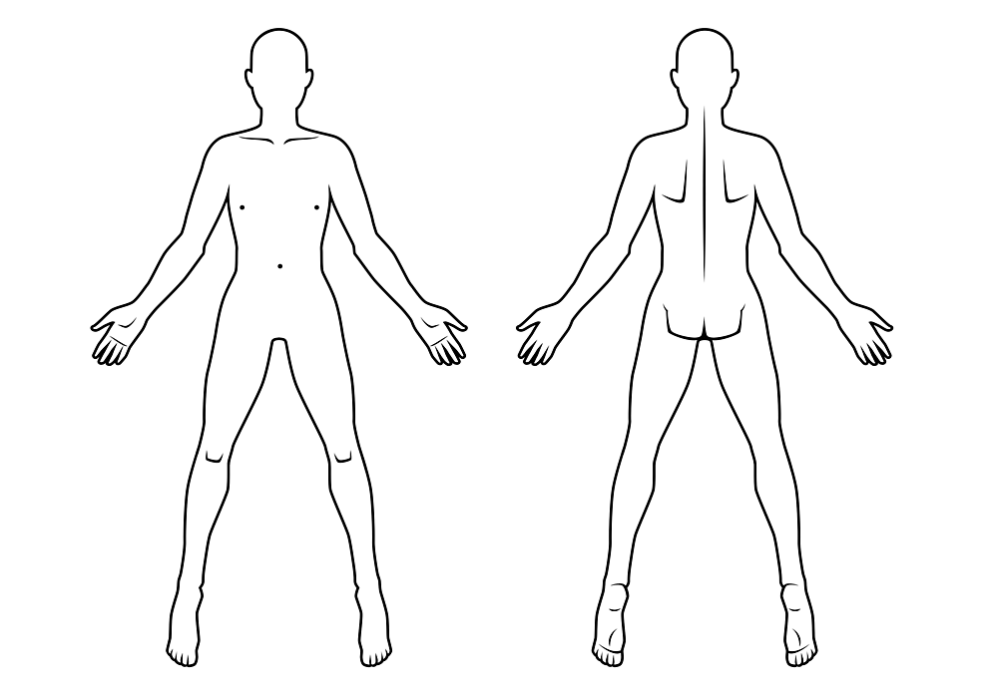
Reference image (body map)

The template digital BM image (reference image (RI)) (Figure 1) has been designed to be genderless to provide easily recognizable body parts to deliver most of the body areas for sketching and the frontal and back body areas to be of exactly the same size (number of pixels). The RI is in high definition, counting 1.517.036 pixels for the total area of the presented body sides and follows the Rule of Nines for Burn. Finally, the RI has been drawn without antialiasing, meaning that it has as less gray tones as possible, and therefore, the boundaries of the body shape are recognized easier from the analysis software without significant errors coming from gray color areas (error of a few pixels on a 1.5 m pixel image).

The custom-built software was developed in VC++ and designed to use exclusively the aforementioned RI in order to automate its calculations. The Pain Distribution application has the following main properties and functions.

Pain extent

The Pain Distribution application automatically calculates the number of pixels the patient has chosen with the color pen and compares this number with the number of pixels of the whole-body areas. The application calculates the drawn areas by the patient that are only inside the boundaries of the body map and excludes any drawings that happened to exceed the aforementioned limits. For visual inspection and verification, the area taking part in the calculations is automatically painted by the software with the complementary color used by the patient.

Smart Selected Area Calculations tool

When patients, during the drawing procedure, leave some uncolored areas that they intended to color, by selecting the “Use Smart Selected Area Calculations” option, these areas are covered with color and take part in the calculations (filling the uncolored gaps inside the drawings).

Probe tool

**Figure 2:**
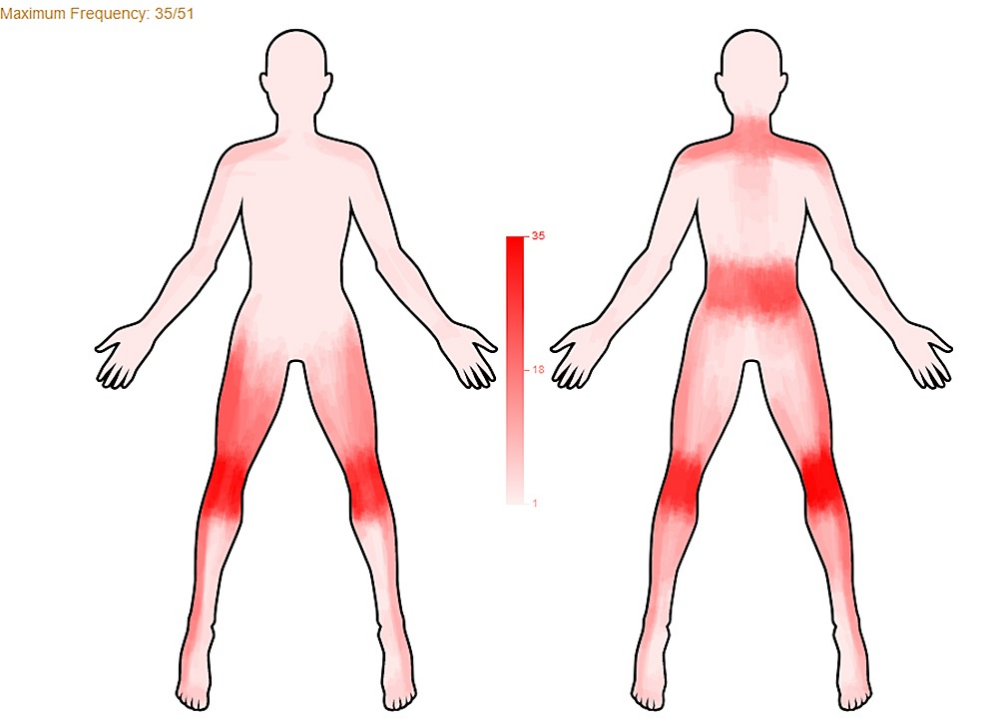
Frequency distribution image

This tool provides the frequency distribution from a group of images (Figure 2). Α color grid indicates the percentage of individuals that reported pain in a specific area.

Central Sensitization threshold

Prior to any calculation, the user has the option to select the threshold over which the patient is considered with Central Sensitization [29,30].

Results

The results are presented in the Graphical User Interface (GUI) for both every image and the frequency distribution, providing mean values, standard deviations, and CS indicators according to the Central Sensitization threshold chosen by the user. The Pain Distribution application has the ability to export the results in *.txt format (Table 1) for further statistical or other calculations in a software of choice.

**Table 1:** *.txt format file exported by the software with all the information for further analysis

#	Filename	Ratio (%)	CS
1	Name 1.png	11.93	False
2	Name 2.png	12.22	False
3	Name 3.png	18.5	True
4	Name 4.png	10.84	False
…	…	…	…
50	Name 50.png	10.54	False
51	Name 51.png	21.2	True
CS threshold = 16%			
Total number of pictures = 51			
Number of pictures with CS = 7 (13.73%)			
Mean picture ratio = 12.71			
Mean picture with CS ratio = 31.42			
Mean picture without CS ratio = 9.73			
SD picture ratio = 5.94			
SD picture with CS ratio = 19.98			
SD picture without CS ratio = 3.22			
Frequency distribution of the whole picture set = 35/51			
Frequency distribution of the CS picture set = 7/7			
Frequency distribution of the non-CS picture set = 28/44			

## Discussion

The Pain Distribution application and the reference image developed in the present project should be used according to the following procedures.

Patients must use a tablet with a drawing application of the operator’s choice, such as SketchBook Pro (Autodesk, San Rafael, CA, USA) and with the reference image preloaded. The RI is stored permanently inside the Pain Distribution application and can be exported as a Reference.png file at any time, for transferring to other applications or devices (i.e., a tablet). Patients are then requested to color with a stylus the area where they feel pain. The files are then saved and transferred from the tablet to the Pain Distribution application for analysis.

Any color can be used with the stylus but black and, obviously, white, while the type and size of the pen stroke do not affect the calculations. However, for a group of patients participating in a research project, it is advised to use the same color in order to produce visually correct frequency distribution images.

The validity and reliability of the software are absolute: the Pain Distribution application follows the same procedures in the built-in code constantly and is free from bugs and viruses. However, additional reliability and validity trials are required, from the clinical point of view, to clarify errors that may arise from the patient, researcher, or the procedure. More detailed instructions about the use of the software and the detailed results of possible errors related to the RI are given in the built-in “Pain Distribution App Guide.” 

The limitation of the software is that it only calculates the location and extent of the pain and not its amplitude or other pain characteristics such as deep pain sensation. Depending on scientific grounds, as well as on the demands of the pain scientific community, these or additional features may be added in a future edition.

## Conclusions

In the Department of Physiotherapy, University of Thessaly, Greece, we have recently designed and developed a software for pain drawing analysis, named “Pain Distribution” application, which can be used in both clinical work and academic research. To our knowledge, there is no other application available for that purpose. The software has many novel features and tools, as it can calculate pain extent by automatically excluding the areas drawn outside the body map, provides the frequency distribution from a group of images to graphically represent the most common chosen areas of pain, gives an option to select a Central Sensitization threshold, and exports the results for further analysis. Additionally, the software is user-friendly, needs no prior knowledge, and includes an analytical User’s Guide for installation and use.

The Pain Distribution application and its reference image are free for any use by mentioning the contributors. In case of use in research/academic, citing the present paper would be highly appreciated. The software (pain_distribution.exe), together with some dummy images and the Application Guide in pdf format, can be downloaded at https://www.hprl.physio.uth.gr/pain-distribution.
